# Personality-related and psychosocial correlates of sick leave days in Germany during the COVID-19 pandemic: findings of a representative survey

**DOI:** 10.1186/s13690-022-00980-6

**Published:** 2022-11-04

**Authors:** André Hajek, Hans-Helmut König

**Affiliations:** Department of Health Economics and Health Services Research, University Medical Center Hamburg-Eppendorf, Hamburg Center for Health Economics, Hamburg, Germany

**Keywords:** Sick leave days, Sickness absence, Personality, Psychosocial, Loneliness, Empathy, Altruism, COVID-19, Coronavirus anxiety

## Abstract

**Background:**

The aim of our study was to assess the personality-related and psychosocial correlates of sick leave days in Germany during the COVID-19 pandemic.

**Methods:**

We used data from a representative online-survey covering the general German adult population (data collection: mid-March 2022). We restricted our sample to full-time employed individuals aged 18 to 64 years (n = 1,342 individuals). Sick leave days in the preceding 12 months served as outcome measure. Validated and established tools were used to quantify personality characteristics and psychosocial factors (such as the Coronavirus Anxiety Scale or the De Jong Gierveld loneliness tool). Negative binomial regression models were used.

**Results:**

After adjusting for various sociodemographic and health-related factors, regressions showed that a higher number of sick leave days was associated with lower levels of conscientiousness (IRR: 0.84, 95% CI: 0.73-0.97), higher levels of openness to experience (IRR: 1.19, 1.04–1.35), less coronavirus anxiety (IRR: 0.90, 95% CI: 0.86-0.93), and more depressive symptoms (IRR: 1.06, 1.02–1.11).

**Conclusion:**

After adjusting for various sociodemographic and health-related factors, our study showed an association between personality-related and psychosocial factors with sick leave days. More research is required to clarify the underlying pathways.

**Supplementary Information:**

The online version contains supplementary material available at 10.1186/s13690-022-00980-6.

## Introduction

Sick leave days reflect an under-utilization of an individual’s capital to create gross domestic product [1]. Thus, they are important for the society as a whole. Moreover, sick leave days (and particularly long-term absenteeism) can reduce household income and can increase the risk of future sick leave [2]. Hence, knowledge about the factors associated with sick leave days is of great importance.

Previous studies mainly focused on rather ‘classical’ correlates of sick leave days such as sociodemographic factors [2] – showing that particularly older age can contribute to sick leave [2]. Only a few studies have examined personality-related and psychosocial correlates of sick leave days [1, 3–8]. Moreover, these studies mainly focused on times prior to the pandemic. Therefore, the aim of our study was to clarify the personality-related and psychosocial correlates of sick leave days in Germany during the pandemic. Such knowledge may be of importance to address individuals at risk for a high number of sick leave days.

During times of the pandemic, factors such as empathy (i.e., ability to imagine what life is like for another individual [9]), altruism (i.e., selflessness [10]) or agreeableness (i.e., the extent to which a person is cooperative and friendly [11]) may be of high importance for sick leave days. For instance, individuals scoring high in agreeableness may try to avoid an argument, for example, when working in the office with a cold during the COVID-19 pandemic (and working at home is impossible). Furthermore, individuals scoring high in empathy may have a good ability to imagine what life is for other individuals (e.g., for other individuals with certain chronic conditions who are at risk for a severe course of COVID-19). Similarly, individuals scoring high in altruism often help others and put the needs of others above their own. They actively care about the quality of life of others [10]. For example, higher altruism is associated with a higher likelihood of vaccination against COVID-19 [12]. In sum, we assume that individuals scoring high in empathy and altruism may try to avoid an infection (e.g., with COVID-19) so as not to pose a risk to work colleagues. Thus, due to this cautious behavior they may have a lower number of sick leave days. Moreover, psychosocial factors such as coronavirus anxiety, loneliness or perceived social isolation may be of importance for sick leave days during the pandemic. For instance, a high coronavirus anxiety may also partly reflect a cautious approach to potential infections and may thus contribute to a lower number of sick leave days. Additionally, individuals who are lonely may have a higher number of sick leave days due to the association between loneliness and well-being [13].

## Materials and methods

### Sample

The current survey drew on data from a nationally representative online survey of Germans aged 18 to 74 (where 3,091 respondents participated). A key aim of this study was to clarify the determinants of healthcare use and sick leave days. In this current study, we restricted our sample to full-time employed individuals aged 18 to 64 years (n = 1,342). The age restriction was made because individuals aged 65 years and over are commonly retired in Germany. Moreover, we focused on full-time employed individuals to ease the comparison. In our view, it is rather difficult to compare the number of part-time employed individuals (e.g., when one individual is working 5 h a week and another individual is working 30 h a week).

The survey was carried out in mid-March 2022. The market research firm Bilendi & respondi – an ISO 26,362 certified online sample provider – recruited participants using its own actively managed online access panel. The participants were rewarded by bilendi & respondi based on their Mingle points system. The points awarded were nominal in value, as a small compensation for the time it took to complete the survey.

Respondents were drawn from an online sample in such a way that their age, gender, and federal state distribution were representative of the entire German adult population (quota sampling) [14]. About 11,900 individuals were invited to participate. A sample selection bias could not be calculated for reasons of data availability.

All individuals provided informed consent. This study was approved by the University Medical Center Hamburg-Center Eppendorf’s Local Psychological Ethics Committee (LPEK-0412).

### Outcome

Respondents self-reported the number of sick leave days in the preceding 12 months. The individuals were instructed as follows: “Please indicate all days, not only those for which you have received a doctor’s certificate of incapacity for work”.

This is a common assessment of sick leave days. For example, it is in accordance with the assessment used in the German Socio-Economic Panel (GSOEP) [15] – a well-known and long-running household panel.

### Independent variables

With regard to personality-related factors, we included these factors: The 10-item Big Five Inventory (BFI-10) [16] was used to quantify personality (i.e., agreeableness, conscientiousness, extraversion, neuroticism and openness to experience). It is an established tool to quantify the key personality characteristics (two items per dimension; each dimension goes from 1 to 7, higher values reflect a more pronounced personality factor). Moreover, altruism was quantified using the subscale ‘altruism’ of the International Personality Item Pool (IPIP-5F30F-R1 [17]) consisting of six items (ranging from 1 to 5 in each case). By averaging the recoded items, a score was calculated (1 to 5; higher scores indicate higher altruism). Based on the Interpersonality Reactivity Index (IRI [9]; German version: Saarbrucken personality questionnaire, SPF [18]; called SPF-K ([19]), empathy was measured. It has four items (5 levels in each case). Following Paulus [19], a sum score was created (ranging from 4 to 20, higher values reflect higher empathy). Further details are provided by Paulus [19].

With regard to psychosocial factors, we included these factors: Loneliness was quantified using the 6-item De Jong Gierveld loneliness tool) [20]. It consists of six items. By averaging the items, a loneliness score was computed (from 1 to 4; higher values reflect higher levels of loneliness). Perceived social isolation was quantified based on the Bude and Lantermann [21] tool which has four items. A score was created by averaging the items (from 1 to 4, with higher values reflecting higher perceived social isolation). Coronavirus anxiety was measured using the coronavirus anxiety scale [22–24]. It has five items. A sum score was computed (from 0 to 20, higher values reflect higher coronavirus anxiety). Moreover, the Patient Health Questionnaire-9 (PHQ-9) was used to assess depressive symptoms. It consists of nine items (sum score ranges from 0 to 27, with higher values corresponding to more depressive symptoms) [25]. To assess anxiety symptoms, The Generalized Anxiety Disorder-7 (GAD-7) [26] was used. It has seven items (sum score ranges from 0 to 21, with higher values reflecting more anxiety symptoms).

### Covariates

Based on prior research (e.g., [27–29] and based on theoretical considerations, covariates were selected. More precisely, as covariates, we included several sociodemographic and health-related factors in regression analysis: Age, sex (three categories, reference category: men; women; diverse), one or more children in own household (reference category: no; yes), family situation (married, living together with spouse; married, not living together with spouse; single; widowed; divorced; dichotomized into: married, living together with spouse; other including all other categories (as reference category)), and school education (reference category: Upper secondary school; Qualification for applied upper secondary school; Polytechnic Secondary School; Intermediate Secondary School; Lower Secondary School; Currently in school training/education; Without school-leaving qualification). Additionally, we included vaccination against Covid-19 (reference category: no; yes), self-rated health (single-item measure ranging from 1 to 5; higher values reflect better self-rated health) and the presence of one or more chronic conditions (no; yes) in regression analysis.

In additional analysis, and in accordance with prior research (e.g., [30, 31]), it was also adjusted for some lifestyle-factors. More precisely, it was adjusted for alcohol consumption (reference category: daily; several times per week; once a week; 1–3 times per month; less often; never), smoking behavior (reference category: yes, daily; yes, sometimes; no, not anymore; never smoker), and frequency of sports activities (reference category: no sports activity; less than one hour a week; regularly, 1–2 h a week; regularly, 2–4 h a week; regularly, more than 4 h a week).

### Statistical analysis

Firstly, sample characteristics are shown. Thereafter, multiple negative binomial regressions were used to examine the personality-related and psychosocial correlates of sick leave days. For example, compared to a Poisson model, a negative binomial regression had much smaller BIC values (Poisson model, BIC: 35,484.5; negative binomial model, BIC: 7,196.3). This shows that the negative binomial model fits our data much better.

The significance level was set at p < 0.05. Stata 16.1 (Stata Corp., College Station, Texas) was used for performing statistical analyses.

## Results

### Sample characteristics

Characteristics of the sample are depicted in Table 1. Average age equaled 43.3 years (SD: 11.6 years; 18 to 64 years) and about 61.1% were male. In sum, 64.4% of the individuals were married, living together with spouse and 33.0% of the individuals had at least one child in their own household. Moreover, 88.5% of the individuals were vaccinated against COVID-19 and 34.4% of the individuals had at least one chronic disease. Overall, the average number of sick leave days was 10.3 (SD: 31.0; median: 0; 75% quartile: 10; interquartile range: 10; ranging from 0 to 365).

**Table 1:** Sample characteristics among full-time employed individuals aged 18 to 64 years (n = 1,342; data collection: mid-March 2022)

Variables	Mean (SD) plus skewness, kurtosis, median, IQR and range / N (%)
Sick leave days	10.3 (31.0); 7.6; 74.5; 0; 10.0; 365.0
Gender	
-Male	820 (61.1%)
-Female	520 (38.7%)
-Diverse	2 (0.1%)
Age	43.3 (11.6); -0.1; 1.9; 43.0; 21.0; 45.0
Children in own household	
-No	899 (67.0%)
-Yes	443 (33.0%)
Marital status	
-Single/Divorced/Widowed/Married, not living together with spouse	478 (35.6%)
-Married, living together with spouse	864 (64.4%)
Education	
-Upper secondary school	609 (45.4%)
-Qualification for applied upper secondary school	145 (10.8%)
-Polytechnic Secondary School	79 (5.9%)
-Intermediate Secondary School	405 (30.2%)
-Lower Secondary School	99 (7.4%)
-Currently in school training/education	4 (0.3%)
-Without school-leaving qualification	1 (0.1%)
Employment status	
-Full-time employed	1342 (100.0%)
-Retired	
-Other	
Chronic diseases	
-Absence of at least one chronic disease	881 (65.6%)
-Presence of at least one chronic disease	461 (34.4%)
Self-rated health (from 1 = very bad to 5 = very good)	3.8 (0.8); -0.6; 3.6; 4.0; 1.0; 4.0
Being vaccinated against COVID-19	
-Not being vaccinated	155 (11.5%)
-Being vaccinated	1187 (88.5%)
Extraversion (BFI-10, from 1 to 7, higher values reflect higher extraversion)	4.0 (1.2); 0.1; 2.8; 4.0; 2.0; 6.0
Agreeableness (BFI-10, from 1 to 7, higher values reflect higher agreeableness)	5.1 (1.0); -0.1; 2.5; 5.0; 1.5; 5.5
Conscientiousness (BFI-10, from 1 to 7, higher values reflect higher conscientiousness)	5.6 (1.1); -0.6; 2.8; 6.0; 1.5; 5.5
Neuroticism (BFI-10, from 1 to 7, higher values reflect higher neuroticism)	2.9 (1.2); 0.5; 2.7; 3.0; 2.0; 6.0
Openness to experience (BFI-10, from 1 to 7, higher values reflect higher openness)	4.9 (1.1); -0.1; 2.7; 5.0; 2.0; 6.0
Empathy (SPF-K, from 4 to 20, higher values reflect higher empathy)	13.1 (2.9); -0.2; 3.5; 13.0; 3.0; 16.0
Altruism (Subscale „Altruism“ of the IPIP, from 1 to 5, higher values reflect higher altruism)	3.4 (0.7); -0.2; 3.4; 3.4; 0.8; 4.0
Coronavirus anxiety (CAS, from 0 to 20, higher values reflect higher coronavirus anxiety)	1.5 (3.2); 2.7; 10.4; 0.0; 1.0; 20.0
Depressive symptoms (PHQ-9, 0 to 27, higher values reflect more depressive symptoms)	5.7 (5.3); 1.2; 4.1; 4.0; 7.0; 27.0
Anxiety symptoms (GAD-7, 0 to 21, higher values reflect more anxiety symptoms)	4.6 (4.6); 1.3; 4.5; 3.0; 6.0; 21.0
Loneliness (De Jong Gierveld Loneliness Scale, 1 to 4, higher values reflect higher loneliness)	2.1 (0.6); 0.1; 2.5; 2.0; 0.8; 3.0
Perceived social isolation (Bude Lantermann Scale, 1 to 4, higher values reflect higher perceived social isolation)	1.9 (0.8); 0.6; 2.5; 2.0; 1.3; 3.0

Notes: Skewness reflects the degree and direction of asymmetry. For example, a normal distribution has a skewness of zero and a left-skewed distribution has a negative skewness. Kurtosis reflects the heaviness of the tails of a distribution. A kurtosis of three reflects a normal distribution. A kurtosis greater than three reflects a heavy tailed distribution

With regard to personality-related factors, average extraversion score was 4.0 (SD: 1.2), average agreeableness score was 5.1 (SD: 1.0), average conscientiousness score was 5.6 (SD: 1.1), average neuroticism score was 2.9 (SD: 1.2), and average openness to experience score was 4.9 (SD: 1.1). Furthermore, average empathy score was 13.1 (SD: 2.9) and average altruism score was 3.4 (SD: 0.7).

With regard to psychosocial factors, average coronavirus anxiety score was 1.5 (SD: 3.2), average depressive symptoms score was 5.7 (SD: 5.3), average anxiety score was 4.6 (SD: 4.6), average loneliness score was 2.1 (SD: 0.6), and average perceived social isolation score was 1.9 (SD: 0.8). Additional details are given in Table 1. It may be worth noting a correlation matrix (using Pearson’s r) for the key variables is provided in Supplementary Table 1 (an additional non-parametric correlation with Spearman’s Rho is shown in Supplementary Table 2).

### Regression analysis

Findings of multiple negative binomial regression analysis are given in Table 2 (complete results including the covariates are shown in Supplementary Table 3). After adjusting for various sociodemographic and health-related factors, regressions showed that a higher number of sick leave days was associated with lower levels of conscientiousness (IRR: 0.84, 95% CI: 0.73-0.97), higher levels of openness to experience (IRR: 1.19, 1.04–1.35), less coronavirus anxiety (IRR: 0.90, 95% CI: 0.86-0.93), and more depressive symptoms (IRR: 1.06, 1.02–1.11). In contrast, the other three Big-Five factors (agreeableness, extraversion and neuroticism), empathy and altruism as well as anxiety symptoms, loneliness, and perceived social isolation were not associated with the number of sick leave days.

**Table 2:** Personality-related and psychosocial correlates of sick leave days. Results of multiple negative binomial regression analysis – based on full-time employed individuals aged 18 to 64 years (data collection: mid-March 2022)

Independent variables	Sick leave days
Personality-related factors	
Extraversion (BFI-10)	1.04
	(0.94–1.15)
Agreeableness (BFI-10)	1.04
	(0.91–1.19)
Conscientiousness (BFI-10)	0.84*
	(0.73–0.97)
Neuroticism (BFI-10)	1.01
	(0.89–1.14)
Openness to experience (BFI-10)	1.19*
	(1.04–1.35)
Empathy (SPF-K)	1.02
	(0.96–1.07)
Altruism (Subscale „Altruism“ of the IPIP)	1.05
	(0.83–1.31)
Psychosocial factors	
Coronavirus anxiety (CAS)	0.90***
	(0.86–0.93)
Depressive symptoms (PHQ-9)	1.06**
	(1.02–1.11)
Anxiety symptoms (GAD-7)	0.98
	(0.94–1.03)
Loneliness (De Jong Gierveld Loneliness Scale)	0.96
	(0.75–1.22)
Perceived social isolation (Bude Lantermann Scale)	0.98
	(0.79–1.21)
Potential confounders	✓
Constant	17.56**
	(2.97–103.80)
Observations	1,342
Pseudo R²	0.02

Incidence Rate Ratios are reported; 95% CI in parentheses; *** p < 0.001, ** p < 0.01, * p < 0.05, + p < 0.10; it was adjusted for sex, age, presence of at least one child in own household, marital status, educational level, employment status, being vaccinated against Covid-19, presence of at least one chronic condition and self-rated health

In additional analysis, it was also adjusted for lifestyle-factors (including smoking status, alcohol intake and frequency of sports activities; see Table 3). However, our findings remained nearly the same in terms of effect size and significance.

**Table 3:** Personality-related and psychosocial correlates of sick leave days. Results of multiple negative binomial regression analysis – based on full-time employed individuals aged 18 to 64 years (data collection: mid-March 2022)

Independent variables	Sick leave days
Personality-related factors	
Extraversion (BFI-10)	1.04
	(0.94–1.15)
Agreeableness (BFI-10)	1.06
	(0.94–1.21)
Conscientiousness (BFI-10)	0.82**
	(0.71–0.93)
Neuroticism (BFI-10)	1.01
	(0.89–1.13)
Openness to experience (BFI-10)	1.20**
	(1.07–1.35)
Empathy (SPF-K)	1.02
	(0.97–1.08)
Altruism (Subscale „Altruism“ of the IPIP)	1.03
	(0.82–1.29)
Psychosocial factors	
Coronavirus anxiety (CAS)	0.90***
	(0.86–0.94)
Depressive symptoms (PHQ-9)	1.05*
	(1.01–1.10)
Anxiety symptoms (GAD-7)	0.98
	(0.94–1.03)
Loneliness (De Jong Gierveld Loneliness Scale)	0.99
	(0.78–1.26)
Perceived social isolation (Bude Lantermann Scale)	0.97
	(0.79–1.19)
Potential confounders	✓
Constant	18.22**
	(3.03–109.67)
Observations	1,342
Pseudo R²	0.02

Incidence Rate Ratios are reported; 95% CI in parentheses; *** p < 0.001, ** p < 0.01, * p < 0.05, + p < 0.10; it was adjusted for sex, age, presence of at least one child in own household, marital status, educational level, employment status, being vaccinated against Covid-19, presence of at least one chronic condition, self-rated health, smoking status, alcohol intake and frequency of sports activities

## Discussion

Based on data from the general adult population in Germany during later stages of the COVID-19 pandemic, our aim was to identify the personality-related and psychosocial correlates of sick leave days. After adjusting for various sociodemographic and health-related factors, regressions showed that a higher number of sick leave days was associated with lower levels of conscientiousness, higher levels of openness to experience, less coronavirus anxiety, and more depressive symptoms. According to previous work which translated relative effect sizes into indices of effect size in public health studies [32, 33], the corresponding IRRs identified in our study can be categorized as small.

With regard to the level of the personality- and psychosocial correlates, our present descriptive findings are very similar compared to prior studies [12, 34, 35]. The average number of sick leave days reported in our study (during later stages of the pandemic) is also quite comparable to the average number of sick leave days reported in former research focusing on Germany prior to the pandemic [3]. Thus, it may be the case that the pandemic did not greatly affect sick leave days among employed individuals in Germany.

Given the fact that higher conscientiousness is associated with several positive lifestyle-related factors such as lower alcohol intake [36], non-smoking [37], higher physical activity [38], use of preventive healthcare [39] - and also with favorable health-related factors (e.g., lower risk for future frailty [40]), the link between higher conscientiousness and a lower number of sick leave days is highly plausible. Moreover, a higher openness to experience reflects an intrinsic desire for experience. Thus, the real life experiences may, among other things, increase the risk of getting infected (e.g., with COVID-19) and may thus explain the association between higher levels of openness to experience and a higher number of sick leave days.

It appears to be plausible that less coronavirus anxiety is associated with a higher number of sick leave days in our study. Lower scores of coronavirus anxiety may (at least partly) reflect a more careless approach to COVID-19 (involving a higher risk to catch any infectious diseases) and an actual previous infection with COVID-19 – which may explain the higher number of sick leave days. Moreover, a higher number of depressive symptoms was associated with a higher number of sick leave days. This supports the bulk of previous studies [41] – and can, among other things, be explained by certain depressive symptoms (e.g., fatigue or insomnia).

Interestingly, factors such as empathy, agreeableness and altruism were not significantly associated with the number of sick leave days. As outlined in the introduction, this is in contrast to our expectations. Such non-significant associations may be explained by the fact that such factors may not be necessarily correlated with health-related behaviors or by the fact that our data collection took place in March 2022 – which reflects a late stage of the COVID-19 pandemic where every individual already had the opportunity to be vaccinated against COVID-19 and the coronavirus variants prevalent in Germany did not usually lead to a severe course.

Moreover, factors such as loneliness and perceived social isolation were also not associated with the number of sick leave days. This may be explained by the fact that it was already adjusted for various sociodemographic, personality-, psychosocial and health-related factors. For example, perceived social isolation is moderately positively correlated with depressive symptoms, anxiety symptoms or neuroticism. Actually, when we only include loneliness and perceived social isolation as independent variables (i.e., without further adjustments), there was a positive association between perceived social isolation and the number of sick leave days (IRR: 1.27, 1.002-1.60).

When interpreting our findings, some strengths and weaknesses are worth acknowledging. Data were drawn from a large, representative study (in terms of age bracket, state and sex). However, it should be noted that the possibility of a sample selection bias cannot be completely ruled out. Moreover, the questionnaire was exclusively available in German language. Thus, some non-native speakers may be ruled out which somewhat limits the generalizability. Additionally, a potential online bias cannot be dismissed.

An established question was used to assess sick leave days. However, the existence of some recall bias cannot be fully dismissed. A former study noted that the prevalence of sick leave days may be somewhat underestimated [42]. However, a more recent study [43] showed a good agreement between self-reported sick leave days and register information on sick leave days. This former study [43] also concluded that “the use of retrospectively collected self-reported sick leave days can be very useful in epidemiological studies” (p. 66). Established and valid tools were used to quantify personality-related and psychosocial factors. Our study has a cross-sectional design which makes it difficult to clarify the directionality between our variables of interest. Thus, future research based on longitudinal data is desirable. Moreover, a bit more complex models could be used (such as zero-inflated negative binomial models). However, the AIC and BIC values were roughly comparable between these models (zero-inflated negative binomial model, AIC: 6,906.1, BIC: 7,171.4; worth repeating for negative binomial model, AIC: 7,055.9, BIC: 7,196.3).

## Conclusion

After adjusting for various sociodemographic and even health-related factors, our study showed an association between personality-related (e.g., conscientiousness) and psychosocial factors (e.g., coronavirus anxiety) with sick leave days. Knowledge about these factors may assist in addressing individuals at risk for a high number of sick leave days. Present sick leave days may have negative economic consequences which in turn can contribute to a lower health in the future [44]. Thus, from a public health perspective, such knowledge is of great importance. More research is required to clarify the underlying pathways.

## Electronic supplementary material

Below is the link to the electronic supplementary material.

Supplementary Material 1

## Data Availability

The datasets used and analysed during the current study are available from the corresponding author on reasonable request for all interested researchers.
