# Supplementary material for: Personality-related and psychosocial correlates of sick leave days in Germany during the COVID-19 pandemic: findings of a representative survey
Source: Arch Public Health. 2022 Nov 4;80:227. doi: 10.1186/s13690-022-00980-6 (PMC9635154; doi:10.1186/s13690-022-00980-6)
Supplement: Supplementary file 1 — Supplementary Material 1 [file 13690_2022_980_MOESM1_ESM.doc]

Supplementary Table 1. Correlation matrix for key variables among full-time employed individuals aged 18 to 64 years (n=1,342; data collection: mid-March 2022)

|  | 1 | 2 | 3 | 4 | 5 | 6 | 7 | 8 | 9 | 10 | 11 | 12 | 13 |
| --- | --- | --- | --- | --- | --- | --- | --- | --- | --- | --- | --- | --- | --- |
| 1. Extraversion | 1.00 |  |  |  |  |  |  |  |  |  |  |  |  |
| 2. Agreeableness | 0.06 | 1.00 |  |  |  |  |  |  |  |  |  |  |  |
| 3. Conscientiousness | 0.11^**^ | 0.34^***^ | 1.00 |  |  |  |  |  |  |  |  |  |  |
| 4. Neuroticism | -0.24^***^ | -0.28^***^ | -0.38^***^ | 1.00 |  |  |  |  |  |  |  |  |  |
| 5. Openness to experience | 0.37^***^ | 0.28^***^ | 0.30^***^ | -0.33^***^ | 1.00 |  |  |  |  |  |  |  |  |
| 6. Empathy | 0.07 | 0.27^***^ | 0.11^**^ | 0.06 | 0.25^***^ | 1.00 |  |  |  |  |  |  |  |
| 7. Altruism | 0.03 | 0.28^***^ | 0.09 | 0.01 | 0.14^***^ | 0.47^***^ | 1.00 |  |  |  |  |  |  |
| 8. Coronavirus anxiety | -0.01 | -0.17^***^ | -0.29^***^ | 0.32^***^ | -0.13^***^ | 0.15^***^ | 0.05 | 1.00 |  |  |  |  |  |
| 9. Depressive symptoms | -0.13^***^ | -0.14^***^ | -0.26^***^ | 0.52^***^ | -0.16^***^ | 0.18^***^ | 0.11^**^ | 0.48^***^ | 1.00 |  |  |  |  |
| 10. Anxiety symptoms | -0.11^**^ | -0.16^***^ | -0.19^***^ | 0.55^***^ | -0.13^***^ | 0.22^***^ | 0.12^***^ | 0.47^***^ | 0.83^***^ | 1.00 |  |  |  |
| 11. Loneliness | -0.25^***^ | -0.24^***^ | -0.22^***^ | 0.39^***^ | -0.19^***^ | -0.06 | -0.11^**^ | 0.24^***^ | 0.46^***^ | 0.44^***^ | 1.00 |  |  |
| 12. Perceived social isolation | -0.24^***^ | -0.24^***^ | -0.34^***^ | 0.50^***^ | -0.26^***^ | 0.05 | -0.02 | 0.39^***^ | 0.57^***^ | 0.55^***^ | 0.62^***^ | 1.00 |  |
| 13. Sick leave days | 0.04 | 0.04 | -0.02 | 0.06 | 0.05 | 0.03 | 0.06 | -0.03 | 0.12^***^ | 0.10^*^ | 0.03 | 0.07 | 1.00 |

Notes: Pearson correlations are shown; *** p<0.001, ** p<0.01, * p<0.05 (Sidak-Holm adjusted significance levels)

Supplementary Table 2. Correlation matrix for key variables among full-time employed individuals aged 18 to 64 years (n=1,342; data collection: mid-March 2022) – non-parametric

|  | 1 | 2 | 3 | 4 | 5 | 6 | 7 | 8 | 9 | 10 | 11 | 12 | 13 |
| --- | --- | --- | --- | --- | --- | --- | --- | --- | --- | --- | --- | --- | --- |
| 1. Extraversion | 1.00 |  |  |  |  |  |  |  |  |  |  |  |  |
| 2. Agreeableness | 0.04 | 1.00 |  |  |  |  |  |  |  |  |  |  |  |
| 3. Conscientiousness | 0.11** | 0.34*** | 1.00 |  |  |  |  |  |  |  |  |  |  |
| 4. Neuroticism | -0.24*** | -0.29*** | -0.41*** | 1.00 |  |  |  |  |  |  |  |  |  |
| 5. Openness to experience | 0.35*** | 0.27*** | 0.31*** | -0.35*** | 1.00 |  |  |  |  |  |  |  |  |
| 6. Empathy | 0.07 | 0.25*** | 0.12** | 0.06 | 0.22*** | 1.00 |  |  |  |  |  |  |  |
| 7. Altruism | 0.04 | 0.28*** | 0.11** | -0.02 | 0.15*** | 0.47*** | 1.00 |  |  |  |  |  |  |
| 8. Coronavirus anxiety | -0.01 | -0.12*** | -0.21*** | 0.32*** | -0.10* | 0.17*** | 0.07 | 1.00 |  |  |  |  |  |
| 9. Depressive symptoms | -0.14*** | -0.13*** | -0.27*** | 0.51*** | -0.15*** | 0.16*** | 0.10* | 0.43*** | 1.00 |  |  |  |  |
| 10. Anxiety symptoms | -0.11 | -0.14*** | -0.20*** | 0.52*** | -0.12*** | 0.22*** | 0.12** | 0.46*** | 0.82*** | 1.00 |  |  |  |
| 11. Loneliness | -0.24*** | -0.23*** | -0.23*** | 0.40*** | -0.20*** | -0.07 | -0.12*** | 0.26*** | 0.46*** | 0.43*** | 1.00 |  |  |
| 12. Perceived social isolation | -0.26*** | -0.24*** | -0.35*** | 0.51*** | -0.27*** | 0.03 | -0.04 | 0.37*** | 0.54*** | 0.54*** | 0.63*** | 1.00 |  |
| 13. Sick leave days | 0.04 | 0.07 | -0.02 | 0.07 | 0.08 | 0.03 | 0.05 | 0.01 | 0.20*** | 0.14*** | 0.03 | 0.06 | 1.00 |

Notes: Spearman’s Rho correlations are shown; *** p<0.001, ** p<0.01, * p<0.05 (Sidak-Holm adjusted significance levels)

Supplementary Table 3. Personality-related and psychosocial correlates of sick leave days. Results of multiple negative binomial regression analysis – based on full-time employed individuals aged 18 to 64 years (data collection: mid-March 2022)

| Independent variables | Sick leave days |
| --- | --- |
| Personality-related factors |  |
|  |  |
| Extraversion (BFI-10) | 1.04 |
|  | (0.94 - 1.15) |
| Agreeableness (BFI-10) | 1.04 |
|  | (0.91 - 1.19) |
| Conscientiousness (BFI-10) | 0.84* |
|  | (0.73 - 0.97) |
| Neuroticism (BFI-10) | 1.01 |
|  | (0.89 - 1.14) |
| Openness to experience (BFI-10) | 1.19* |
|  | (1.04 - 1.35) |
| Empathy (SPF-K) | 1.02 |
|  | (0.96 - 1.07) |
| Altruism (Subscale „Altruism“ of the IPIP) | 1.05 |
|  | (0.83 - 1.31) |
|  |  |
| Psychosocial factors |  |
|  |  |
| Coronavirus anxiety (CAS) | 0.90*** |
|  | (0.86 - 0.93) |
| Depressive symptoms (PHQ-9) | 1.06** |
|  | (1.02 - 1.11) |
| Anxiety symptoms (GAD-7) | 0.98 |
|  | (0.94 - 1.03) |
| Loneliness (De Jong Gierveld Loneliness Scale) | 0.96 |
|  | (0.75 - 1.22) |
| Perceived social isolation (Bude Lantermann Scale) | 0.98 |
|  | (0.79 - 1.21) |
|  |  |
| Sociodemographic factors |  |
|  |  |
| Sex: - Women (Ref.: Men) | 1.54** |
|  | (1.18 - 2.02) |
| Age | 1.00 |
|  | (0.98 - 1.01) |
| Children in own household: - Yes (Reference: No) | 0.97 |
|  | (0.74 - 1.26) |
| Marital status: - Married, living together with spouse (Ref.: Single/Divorced/Widowed/Married, not living together with spouse) | 1.14 |
|  | (0.87 - 1.48) |
| Highest educational degree: - qualification for applied upper secondary school (Ref.: upper secondary school) | 1.07 |
|  | (0.75 - 1.52) |
| - polytechnic Secondary School | 2.34*** |
|  | (1.49 - 3.68) |
| - intermediate Secondary School | 1.44* |
|  | (1.08 - 1.91) |
| - Lower Secondary School | 1.92* |
|  | (1.10 - 3.37) |
| - currently in school training/education | 1.84 |
|  | (0.32 - 10.51) |
| - without school-leaving qualification | 0.00*** |
|  | (0.00 - 0.00) |
| Health-related factors |  |
|  |  |
| Chronic diseases: Presence of at least one chronic disease (Absence of chronic diseases) | 1.35* |
|  | (1.06 - 1.72) |
| Self-rated health (1 = very bad to 5 = very good) | 0.59*** |
|  | (0.48 - 0.72) |
| Vaccinated against Covid-19: Yes (Ref.: No) | 0.78 |
|  | (0.52 - 1.17) |
| Constant | 17.56** |
|  | (2.97 - 103.80) |
|  |  |
| Observations | 1,342 |
| Pseudo R² | 0.02 |

Incidence Rate Ratios are reported; 95% CI in parentheses; *** p<0.001, ** p<0.01, * p<0.05.
